# Retraction: Metformin protects the heart against hypertrophic and apoptotic remodeling after myocardial infarction

**DOI:** 10.3389/fphar.2026.1967824

**Published:** 2026-08-25

**Authors:** Frontiers Editorial Office

The journal retracts the 27 February 2019 article cited above.

Following publication, the publisher identified concerns regarding the reuse of image material from Figure 7A in another article and discrepancies between the published figure and the source images subsequently provided. The provenance of the proposed replacement image and the relationship between the source images and the quantification reported in Figure 7B could not be verified.

The authors were contacted and provided data, but these were not sufficient to explain the discrepancies under investigation. As a result, the data and conclusions of the article are deemed unreliable, and the article has been retracted.

This retraction was approved by the Chief Editors of *Frontiers in Pharmacology* and the Chief Executive Editor of Frontiers. Frontiers would like to thank the concerned reader who contacted us regarding the published article.

